# Loss of the nuclear factor Y subunit B3 gene causes chlorosis and chloroplast development defects in cucumber

**DOI:** 10.1093/hr/uhag138

**Published:** 2026-05-08

**Authors:** Min Sheng, Siqi Wang, Tiefeng Song, Juyong Zhao, Cancan Xia, Huanle He, Junsong Pan, Jian Pan, Haifan Wen

**Affiliations:** College of Horticulture, Shenyang Agricultural University, Shenyang 110866, China; College of Bioscience and Biotechnology, Shenyang Agricultural University, Shenyang 110866, China; Institute of Vegetables, Liaoning Academy of Agricultural Sciences, Shenyang 110161, China; Institute of Vegetables, Liaoning Academy of Agricultural Sciences, Shenyang 110161, China; College of Agriculture and Biology, Shanghai Jiao Tong University, Shanghai 200240, China; Science and Education Service Section, Shanghai Pudong Agrotechnology Extension Center, Shanghai 201201, China; College of Agriculture and Biology, Shanghai Jiao Tong University, Shanghai 200240, China; College of Agriculture and Biology, Shanghai Jiao Tong University, Shanghai 200240, China; College of Horticulture, Shenyang Agricultural University, Shenyang 110866, China; College of Agriculture and Biology, Shanghai Jiao Tong University, Shanghai 200240, China

## Abstract

Chlorophyll, the pigment in plant leaves, is crucial for capturing light during photosynthesis. Mutations that disrupt chlorophyll production or chloroplast development frequently lead to changes in leaf color. Here, we report the identification and characterization of a chlorotic mutant in cucumber (*Cucumis sativus* L.), named *tnyl3*, which exhibits chlorotic cotyledons and seedling lethality. Map-based cloning revealed that the *tnyl3* mutation results from a Tnt1 retrotransposon insertion in a gene encoding *nuclear factor Y subunit B3* (*NF-YB3*), a transcription factor subunit. Compared with the wild type, the mutant exhibited dramatic decreases in chlorophyll a and b levels and a significantly lower net photosynthetic rate. Ultrastructural analysis revealed that the chloroplasts in the *tnyl3* mutants are structurally abnormal and characterized by underdeveloped thylakoid membranes. Gene expression analyses revealed that *CsNF-YB3* is highly expressed in young seedling tissues and is upregulated by light. CRISPR/Cas9 analyses subsequently confirmed that the *tnyl3* phenotype is caused by the loss of *CsNF-YB3*. Yeast two-hybrid and split-LUC assays demonstrated that CsNF-YB3 interacts directly with the cucumber NF-YC2 protein and promotes *CsTIC21* transcription, suggesting that it functions as part of an NF-Y transcriptional complex. Transcriptome profiling of the mutant revealed extensive downregulation of photosynthesis-related genes, which is consistent with its impaired chloroplast function. Our findings establish CsNF-YB3 as a crucial genetic factor for chloroplast development and pigment synthesis in cucumber. This work provides new insight into the NF-Y–mediated regulatory network controlling chloroplast biogenesis and offers a potential genetic target for improving plant photosynthetic performance and vigor.

## Introduction

In higher plants, photosynthesis primarily occurs in foliage, where chlorophyll and carotenoid pigments reside to capture light energy. These pigments are not only critical for plant energy production but also influence the visible appearance and normal growth of leaves [1, 2]. Changes in leaf pigmentation, such as yellowing or variegation, often reflect underlying genetic, developmental, or environmental factors, affecting plant health and productivity [3, 4]. Leaf color mutants therefore provide a valuable model for investigating chlorophyll biosynthesis and chloroplast development mechanisms, highlighting the importance of identifying genetic regulators of pigment accumulation for both crop improvement and basic plant biology.

Leaf variegation, ‘the presence of green and white/yellow patches on leaves’, typically arises from mutations impairing chloroplast biogenesis or pigment synthesis [5]. In *Arabidopsis thaliana*, mutations in genes, such as *VAR1/VAR2* (encoding chloroplast FtsH metalloproteases), cause variegated leaves due to defective plastid maintenance [6]. A point mutation in the *P700 chlorophyll a apoprotein A1* (*PsaA*) gene confers a variegation phenotype in sunflower (*Helianthus annuus*) [7]. In *Cymbidium sinense*, an AP2/ERF family gene (*CsERF2*) was implicated in leaf variegation, indicating a role for hormone signaling in pigment distribution [8]. Disruption of carotenoid biosynthesis can also lead to abnormal plastids and variegated leaves, as observed in *Brassica napus* [9]. In addition to variegation, many mutants with uniformly chlorotic or albino leaves have been characterized [10, 11]. In pepper (*Capsicum annuum*), the yellow leaf mutant *yl1* has been shown to influence chlorophyll content and carotenoid metabolism, revealing the genetic control of leaf coloration in different environments [1].

In cucumber (*Cucumis sativus* L.), investigations of diverse leaf color mutants have revealed numerous nuclear checkpoints that govern chloroplast formation. The archetypal virescent mutant *v-1* is attributable to *CsaCNGC*, a cyclic nucleotide–gated Ca^2+^ channel that initiates greening, whereas the virescent-yellow mutant *CsVYL* encodes a DnaJ-type zinc-finger chaperone that is indispensable for plastid ribosome assembly [4, 12]. A missense change within a tandem *13*-*lipoxygenase* (*13*-*LOX*) cluster perturbs oxylipin-mediated thylakoid construction, yielding a stable yellow-green phenotype, and a single-residue substitution in the histidine–aspartate protein CsHD generates the *yellow*-*young*-*leaf* (*yyl-1*) trait [13, 14]. The photo-bleached, seedling-lethal mutant *tnyl-2*, caused by the loss of the plastid uridylate kinase CsUMPK, depletes the nucleotide pools vital for chloroplast integrity. Persistent yellow *leaf 2.1* (*CsYL2.1*) results from defective plastid triose-phosphate isomerase, while the newly mapped *yl-5* corresponds to a PPR336 homolog essential for organellar RNA metabolism [3, 15, 16]. Collectively, these loci encompass ion transport, protein quality control, lipid signaling, nucleotide synthesis, and carbon metabolism, underscoring the breadth of nuclear pathways influencing chlorophyll accumulation and furnishing valuable targets for cucumber breeding.

While many of the genes underlying these chlorotic or albino mutants encode enzymes or structural components of the chloroplast, less is known about the nuclear regulatory factors that control chloroplast development in cucumber. Nuclear factor Y (NF-Y) transcription factors have emerged as important regulators of light signaling and chloroplast biogenesis in plants [17–19]. NF-Y is a heterotrimeric complex composed of NF-YA, NF-YB, and NF-YC subunits [20]. Certain NF-YC subunits in *Arabidopsis* (e.g. *NF-YC1*, -*YC3*, -*YC4*, and -*YC9*) act in light-regulated development pathways, including influencing photomorphogenesis and repressing brassinolide signaling in the dark [21]. In general, the NF-Y family in *Arabidopsis* is involved in light-mediated gene expression, chlorophyll biosynthesis, and chloroplast development [22–24]. In rice (*Oryza sativa*), plants with reduced expression of *OsNF-YB2* (via antisense or RNA interference) display decreased chlorophyll content and degenerated chloroplasts, and rice *NF-YB2*, *-B3*, and *-B4* have been shown to regulate the expression of several photosynthesis-related genes, such as chlorophyll a/b-binding proteins [25]. Conversely, overexpression of a wheat *NF-YB* gene (*TaNF-YB3*) increases the chlorophyll content, photosynthetic rate, and early plant growth, underscoring the positive role of NF-YB in promoting photosynthetic capacity [26]. These findings suggest that NF-Y transcription factors are critical modulators of chloroplast development across species.

In this study, we isolated a cucumber chlorotic mutant, *tnyl3*, from a Tnt1 retrotransposon insertional mutagenesis library. The *tnyl3* mutant exhibits a striking chlorotic phenotype in seedlings. We performed genetic mapping and identified the causal gene as *CsNF-YB3*, which encodes an NF-YB transcription factor subunit. We further characterized the physiological and cellular defects of the mutants, including their pigment content, photosynthesis rate, and chloroplast ultrastructure. CRISPR/Cas9-mediated knockout assays confirmed that *CsNF-YB3* is associated with the chlorotic phenotype. Yeast two-hybrid (Y2H) and split-LUC assays were used to demonstrate protein–protein interactions between CsNF-YB3 and CsNF-YC2, providing insight into the NF-Y complex in cucumber. In addition, we analyzed the expression pattern of *CsNF-YB3* and conducted RNA sequencing of *tnyl3* and wild-type (WT) seedlings to examine global transcriptional changes. Our findings reveal a crucial role for the *CsNF-YB3* gene in maintaining normal leaf greening and chloroplast development in cucumber and highlight a potential NF-YB/YC regulatory module underlying chloroplast biogenesis. This work not only deepens our understanding of the genetic control of leaf color in plants but also provides a foundation for leveraging chlorophyll-related genes in crop improvement programs.

## Results

### Phenotypic characterization of the *tnyl3* chlorotic mutant

**Figure 1:**
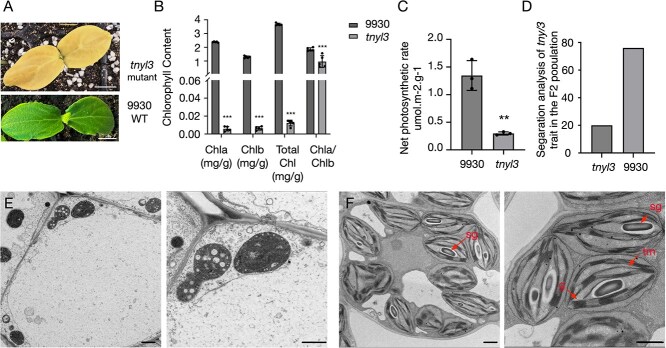
Phenotypic characterization of the *tnyl3* mutant. (A) Seedling phenotypes of WT 9930 and *tnyl3* mutant at 10 days after sowing (DAS). (B) Quantification of chlorophyll a, chlorophyll b, and total chlorophyll content in WT and *tnyl3* cotyledons. Data represent means ± SD (*n* = 5); asterisks indicate significant differences from WT (^***^P < 0.001, Student's *t*-test). (C) Net photosynthetic rates of WT and *tnyl3* seedlings measured on first true leaves, highlighting a substantial decrease in mutant plants. Error bars indicate standard deviations (^**^P < 0.01, Student's *t*-test). (D) Segregation analysis of the chlorotic phenotype in an F_2_ family derived from a cross between *tnyl3* and WT. Green and chlorotic seedlings indicate segregation consistent with single recessive gene inheritance. (E-F) TEM images illustrating chloroplast ultrastructure in *tnyl3* (E) and WT (F) cotyledon cells. WT chloroplasts show typical grana (g), extensive stroma thylakoid membranes (tm) and starch granules (sg). In contrast, mutant chloroplasts exhibit irregular shapes with reduced and disorganized thylakoids. Scale bars: 1 μm.

A chlorotic cucumber mutant named *tnyl3* was identified in the M_1_ segregating population of a Tnt1 insertional mutant library derived from the cucumber inbred line 9930. Phenotypically, compared with the dark-green cotyledons of WT 9930, tnyl3 seedlings displayed distinct chlorotic cotyledons (Fig. 1A). To quantify the severity of chlorosis, the chlorophyll content was measured. The chlorophyll a and b contents in *tnyl3* cotyledons were significantly reduced, representing only approximately 2% to 3% of the levels found in WT plants (Fig. 1B). Consistent with this chlorophyll deficiency, *tnyl3* mutants exhibited a markedly reduced photosynthetic capacity compared with WT plants, with net photosynthetic rates at less than one-third of those in WT plants (Fig. 1C).

Ultrastructural analysis using transmission electron microscopy (TEM) revealed dramatic differences in chloroplast morphology between mutant and WT cotyledons. WT chloroplasts displayed typical ellipsoid shapes with well-developed, densely packed grana stacks interconnected by extensive thylakoid membranes (Fig. 1E). In contrast, *tnyl3* chloroplasts were irregularly shaped and exhibited severely disrupted internal structures with sparsely arranged and fragmented thylakoids. Additionally, the mutant chloroplasts contained fewer grana and lacked the organized arrangement observed in the WT, which correlated with their reduced chlorophyll content and impaired photosynthetic performance (Fig. 1F). The growth potential of the *tnyl3* mutant was subsequently tested under sugar-supplemented conditions. On the sugar-supplemented medium, the *tnyl3* mutant presented an increase in lateral roots and light-yellow cotyledons, and it grew for a slightly extended period, even producing one true leaf (Supplementary Fig. S1). Collectively, these phenotypic and ultrastructural observations clearly indicate that the *tnyl3* mutation causes severe chlorosis by profoundly affecting chlorophyll biosynthesis and chloroplast development.

### Mapping of *tnyl3* and identification of the candidate *CsNF-YB3* gene

**Figure 2:**
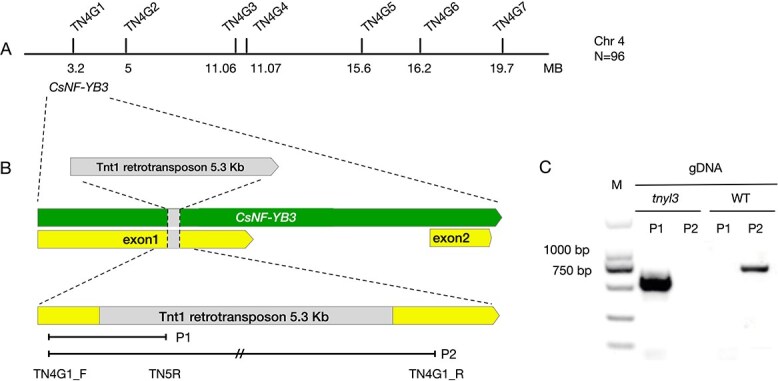
Genetic mapping of *tnyl3* and identification of the *CsNF-YB3* gene. (A) Mapping of the *tnyl3* locus to a region on chromosome 4 using F₂ populations. The graph depicts a section of chromosome 4 with molecular markers; the *tnyl3* locus is confined to an ~3.2-Mb interval (gray box) flanked by markers (shown with their genetic distances). (B) Gene model of *CsNF-YB3* with exons (yellow boxes). The position of the Tnt1 retrotransposon insertion in the *tnyl3* mutant is indicated (gray boxes), which disrupts the exon of *CsNF-YB3*. (C) Cosegregation analysis of the Tnt1 insertion. PCR genotyping was performed on *tnyl3* mutant and WT: Lane M, DNA marker. P1: The presence of a specific ~0.7-kb band indicates the Tnt1 insertion allele. P2: The presence of a specific ~0.5-kb band indicates that the gDNA was complete.

Leveraging the Tnt1 insertional mutant library, we screened all known Tnt1 insertion sites in the TN139 background using 96F₂ individuals. Among the seven loci assayed on chromosome 4 (TN4G1–TN4G7), only TN4G1 exhibited complete genetic linkage with the chlorotic phenotype (Fig. 2A). PCR amplification confirmed the insertion of a 5.3-kb Tnt1 retrotransposon within the exon of *nuclear transcription factor Y subunit B-3* (CsaV4_4G000505 or *CsNF-YB3*), disrupting its open reading frame (Fig. 2B). To further validate this linkage, we expanded the F₂ population to 288 seedlings. Genotyping of all insertion sites revealed that only the CsNF–YB3/TN4G1 locus exhibited a consistent diagnostic pattern—homozygous for the Tnt1 insertion in the *tnyl3* mutant—while the other loci segregated independently (Fig. 2C). These results conclusively identify *CsNF-YB3* as the causal gene for the *tnyl3* recessive chlorotic phenotype.

### Phylogenetic and expression analyses of *CsNF-YB3*

**Figure 3:**
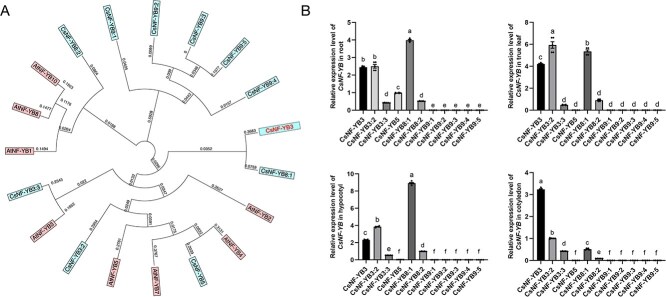
Phylogenetic relationships and expression profile of *CsNF-YB3*. (A) Phylogenetic tree of NF-YB proteins from cucumber and *Arabidopsis*. The tree was constructed using the neighbor-joining method (1000 bootstrap replicates). The red boxes represent *Arabidopsis* NF-YB members, and the blue boxes represent cucumber NF-YB members. The numbers represent genetic distances. (B) Expression levels of *CsNF-YB* members in four seedling organs, as measured by qRT-PCR. The bars represent the means ± SDs (n = 5). Different lowercase letters above the bars indicate significant differences (^**^P < 0.001, Tukey's test).

To place CsNF-YB3 in an evolutionary and family-wide context, we constructed a phylogenetic tree using cucumber NF-YB proteins together with representative *Arabidopsis* NF-YB members (Fig. 3A). Notably, CsNF-YB3 clustered most closely with CsNF-YB8:1, whereas the two additional cucumber genes annotated as NF-YB3-like members (CsNF-YB3:2 and CsNF-YB3:3) were positioned more distantly in the tree, suggesting appreciable divergence within the NF-YB3-related group.

We then examined tissue expression patterns by RT-qPCR across four seedling organs (cotyledon, true leaf, hypocotyl, and root; Fig. 3B). *CsNF-YB3* showed the highest transcript abundance in cotyledons, suggesting that it plays a potential role during early postgermination development when chloroplast biogenesis is initiated. In contrast, *CsNF-YB8:*1 exhibited relatively low expression in cotyledons but became the predominant *NF-YB* transcript in hypocotyls and roots (and remained high in later-developing tissues), indicating a temporally shifted expression program that is biased toward later vegetative organ development. Only *CsNF-YB3* expression was induced by light; plants grown in darkness maintained basal transcript levels, but those exposed to light presented ~2.5-fold elevated expression (Supplementary Fig. S2). Together, the phylogenetic placement and stage/organ-biased expression profiles suggest functional diversification among closely related cucumber *NF-YB* genes and provide a coherent basis for their nonredundant contributions across development.

### Functional verification of *CsNF-YB3* by the CRISPR/Cas9 system

**Figure 4:**
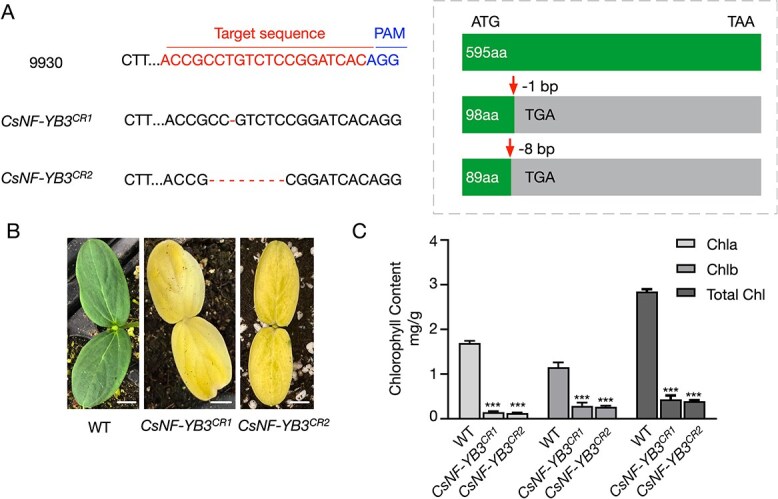
*CsNF-YB3* mutants displayed a chlorotic phenotype in cucumber. (A) Mutation forms of two homozygous *CsNF-YB3* mutants mediated via the CRISPR/Cas9 system. Target sequences and PAM sites are indicated below the red and blue lines. Red dashes represent base deletions, and deletion nucleotides are marked as a minus above the sequence. The dashed box on the right shows the corresponding prematurely terminated protein. The green boxes indicate the CsNF-YB3 protein coding region, and the gray boxes indicate missense sequences resulting from frameshift mutations. (B) Representative images of WT and *CsNF-YB3^CR^* plants at 5 days after sowing. Scale bars: 1 cm. (C) Chlorophyll contents in the WT cucumber and *CsNF-YB3* knockout mutants. The error bars represent the means ± SDs (n = 3). Asterisks indicate significant differences (^**^P < 0.001, Tukey's test).

To confirm that the role of *CsNF-YB3* was related to the chlorotic phenotype, we used the CRISPR/Cas9 system to generate a *CsNF-YB3*-knockout line from WT plants. Two homozygous knockout mutants harboring 1- or 8-bp deletions at the sgRNA target site were obtained. Both mutations led to a premature stop codon and generated a truncated protein (Fig. 4A). The seedlings of the two *CsNF-YB3^CR^* knockout lines presented a chlorotic phenotype, and the markedly stunted growth of these lines was similar to that of the *tnyl3* mutant (Fig. 4B). The chlorophyll a and b contents in the cotyledons of the *CsNF-YB3^CR^* knockout lines were significantly lower than those in 9930 (Fig. 4C). On the basis of these results, we confirmed that the loss of function of *CsNF-YB3* was responsible for the chlorotic phenotype of the *tnyl3* mutant.

### CsNF-YB3 interacts with CsNF-YC2

**Figure 5:**
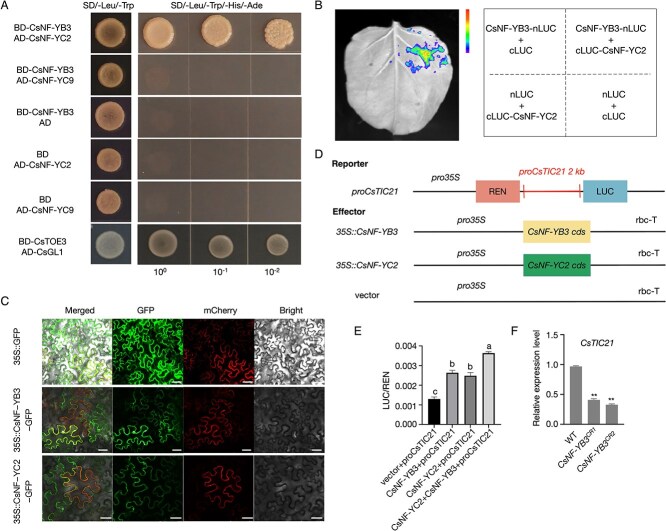
Interaction between CsNF-YB3 and CsNF-YC2. (A) Y2H assay for determining the interaction between CsNF-YB3 and CsNF-YC2. Yeast cotransformants carrying BD-CsNF-YB3 and AD-CsNF-YC2 were grown on selective SD/-Ade/-His/-Trp/-Leu media, whereas the negative controls (BD-CsNF-YB3 with an empty AD vector and an empty BD vector with AD-CsNF-YC2) did not grow. These findings confirm a specific interaction between CsNF-YB3 and CsNF-YC2 in yeast. (B) Image of the fluorescence intensity assay of recombinant LUC catalyzed by the substrate D-luciferin potassium salt obtained from the interaction between the fusion proteins CsNF-YB3-nLUC and cLUC-CsNF-YC2; the chromatogram represents the range of fluorescence intensity. Images were digitally extracted for comparison. (C) Subcellular localization of CsNF-YB3 and CsNF-YC2. Confocal micrographs of tobacco leaf epidermal cells transiently expressing CsNF-YB3-GFP and CsNF-YC2–GFP fusions . Scale bars = 50 μm. (D) In the dual-LUC reporter analysis, *proCsTIC21::*LUC was used as the reporter, and 35S::CsNF-YB3, 35S::CsNF-YC2 and the empty vector were used as the effectors. (E) Dual-LUC reporter analysis. The data indicated that CsNF-YB3 activated the expression of *CsTIC21*, and the empty vector was used as the control. The values are the means ± SDs (n = 6). Different lowercase letters above the bars indicate significant differences (*P* < 0.01, Tukey's test). (F) Expression levels of *CsTIC21* in *CsNF-YB3^CR^* knockout plants. The values are the means ± SDs (n = 3). Asterisks indicate significant differences (^**^P < 0.01, Tukey's test).

To determine whether *CsNF-YB3* functions as part of an NF-YC transcriptional complex, we examined its subcellular localization and interactions with its candidate NF-YC partners. Y2H assays were conducted to test interactions between CsNF-YB3 and two light-responsive NF-YC subunits, CsNF-YC2 and CsNF-YC9. The cotransformation of BD-CsNF-YB3 with AD-CsNF-YC2 enabled yeast growth on stringent selection media (SD/-Ade/-His/-Trp/-Leu), whereas BD-CsNF-YB3 with AD-CsNF-YC9 showed no or negligible interaction (Fig. 5A). Notably, among the closely related CsNF-YB paralogs tested, only CsNF-YB3 showed detectable interactions with CsNF-YC2, whereas CsNF-YB3:2, CsNF-YB3:3 and CsNF-YB8:1 did not interact with CsNF-YC2 under the same conditions (Supplementary Fig. S3). The interaction between CsNF-YB3 and CsNF-YC2 was further confirmed in planta using split-LUC assays. The results revealed that the combination of pC1300-CsNF-YB3-nLUC and pC1300-cLUC-CsNF-YC2 in tobacco produced a LUC signal, whereas the combinations of the empty pC1300-cLUC vector plus pC1300–CsNF-YB3-nLUC and the empty pC1300-nLUC vector plus pC1300-cLUC-CsNF-YC2 produced no LUC signal in the controls (Fig. 5B). Transient expression of CsNF-YB3-GFP and CsNF-YC2-GFP fusions in *Nicotiana benthamiana* leaf epidermal cells revealed that CsNF-YB3 was localized to the cell membrane and nucleus, whereas CsNF-YC2 was localized to the cell membrane (Fig. 5C). Moreover, we observed that CsNF-YB3 and CsNF-YC2 colocalized to the membrane and nucleus, suggesting that CsNF-YC2 is translocated into the nucleus by CsNF-YB3 (Supplementary Fig. S4). These results indicate a strong and specific interaction between CsNF-YB3 and CsNF-YC2.

Recent studies have shown that mutations in *TIC21* also result in a chlorotic phenotype in cucumber [27]. To explore whether the interaction between CsNF-YB3 and CsNF-YC2 influenced *CsTIC21* expression, we performed a dual-LUC assay and found that the expression of the CsNF-YB3 protein led to an increased LUC/REN ratio of *proCsTIC21*, which was further enhanced by the coexpression of the CsNF-YB3 and CsNF-YC2 proteins (Fig. 5D and E). Furthermore, these findings revealed that *CsTIC21* expression was downregulated in the *CsNF-YB3^CR^* knockout line (Fig. 5F). Collectively, these results demonstrate that CsNF-YB3 forms a nuclear NF-YB/YC heterodimer with CsNF-YC2, likely constituting the core complex required for the CCAAT box–mediated activation of the *CsTIC21* gene involved in chloroplast development.

### Transcriptomic impacts of the *tnyl3* mutation

**Figure 6:**
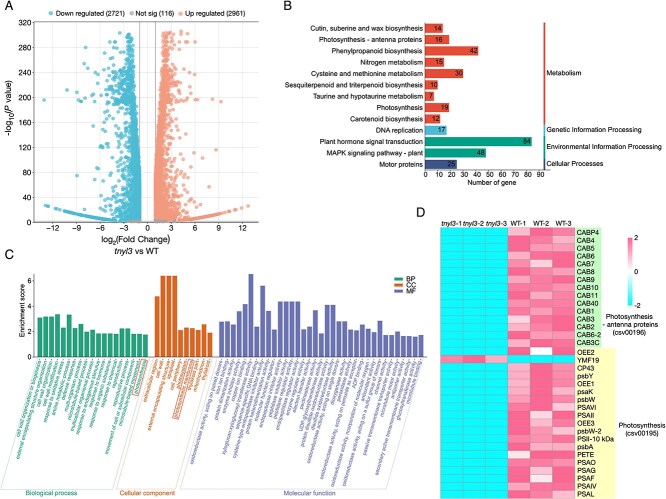
Transcriptomic effects of the *tnyl3* mutation. (A) Volcano plot of DEGs for the WT and the *tnyl3* mutant. (B) KEGG pathways enriched for the DEGs in the *tnyl3* mutation group compared with those in the WT group. (C) GO terms were categorized into biological processes, cellular components and molecular functions on the basis of the number of DEGs. (D) Heatmap of selected photosynthesis-related genes showing strong repression of CAB/LHCB, photosystem I/II core subunits, and chloroplast ribosomal proteins in *tnyl3* compared with those in the wild type. The color scale indicates log₂ expression levels.

To elucidate the downstream effects of *CsNF-YB3* disruption, transcriptome profiling was performed on the cotyledons of *tnyl3* and WT seedlings (three biological replicates per genotype). A total of 5682 differentially expressed genes (DEGs) were identified (|log₂FC| ≥ 1, FDR < 0.01), comprising 2721 downregulated and 2961 upregulated genes (Fig. 6A). GO term analysis revealed significant enrichment of several biological processes related to photosynthesis and cellular components related to the photosynthetic membrane. KEGG pathway analysis revealed high enrichment of plant hormone signal transduction, the MAPK signaling pathway-plant and phenylpropanoid biosynthesis (Fig. 6B and C). Notably, the expression levels of transcripts encoding chlorophyll a/b-binding proteins (CAB/LHCB family) decreased drastically to 10% to 30% of those in the WT. A heatmap of representative photosynthesis-related genes confirmed the widespread suppression of key light-harvesting complex proteins, photosystem I/II subunits, RuBisCO small subunits, and chlorophyll biosynthetic enzymes (Fig. 6D). In parallel, plastid ribosomal protein genes were significantly downregulated, suggesting a compromised chloroplast translation capacity.

In contrast, genes involved in stress responses and hormone signaling were strongly induced in *tnyl3*. The upregulated DEGs included oxidative stress markers and ABA-responsive genes, reflecting a compensatory stress response triggered by impaired chloroplast function. Together, these transcriptomic changes indicate that *CsNF-YB3* is essential for sustaining the nuclear expression programs required for chloroplast biogenesis and photosynthetic competence. The global repression of photosynthetic and plastid ribosomal genes explains the chlorotic, photosynthetically deficient phenotype of *tnyl3*, whereas the induction of stress-related pathways represents a secondary response to chloroplast dysfunction.

## Discussion

This study identifies *CsNF-YB3* as a pivotal nuclear regulator of chloroplast development and photosynthetic capacity in cucumber, providing mechanistic insights into the genetic control of leaf greening. The Tnt1 insertion in the exon of *CsNF-YB3* generated a truncated, likely nonfunctional protein, and genetic analysis confirmed that the disruption of this single recessive gene is sufficient to cause the *tnyl3* chlorotic phenotype. Phenotypically, *tnyl3* seedlings displayed chlorotic cotyledons, a significantly reduced chlorophyll content, stunted growth, and malformed chloroplasts with poorly organized thylakoid membranes, indicating that *CsNF-YB3* is essential for photoautotrophic seedling development.

Our findings align with and extend previous research in other plant species. In rice and *Arabidopsis*, NF-YB subunits are known to activate photosynthesis-related genes and maintain chloroplast integrity, and their silencing leads to similar decreases in chlorophyll content and defective chloroplasts. The transcriptomic data of *tnyl3* further support this conserved role: over 2700 genes were downregulated, including CAB/LHCB light-harvesting proteins, photosystem I/II subunits, and chloroplast ribosomal proteins, whereas more than 2900 stress- and hormone-responsive genes were upregulated, reflecting the retrograde signaling typical of chloroplast dysfunction. This transcriptional pattern parallels that observed in other cucumber mutants, such as *tnyl-2*, indicating that chloroplast defects trigger similar stress responses across different genetic lesions.

**Figure 7:**
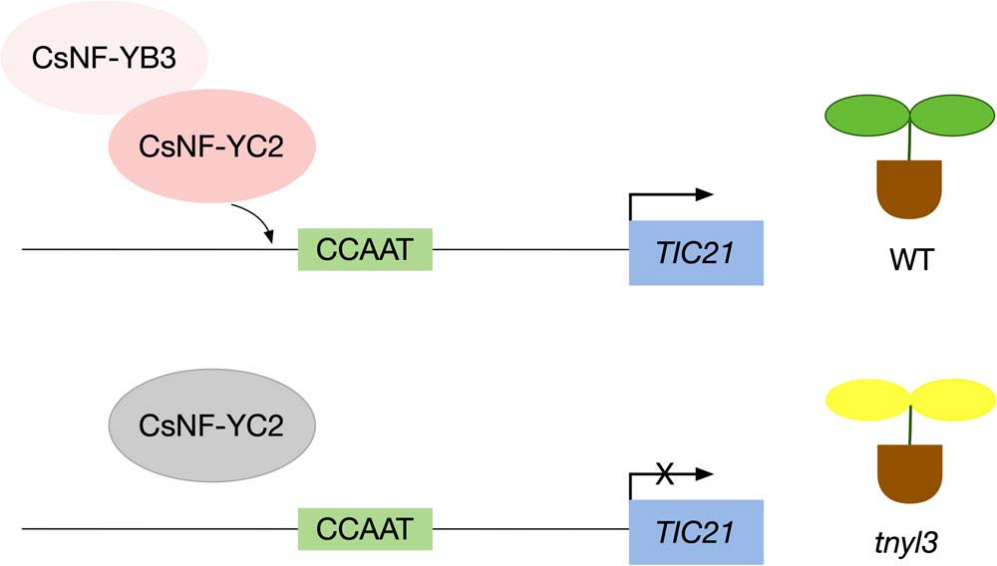
A model chloroplast development in the WT and *tnyl3* mutants. In the WT, CsNF-YB3 interacts with the light-responsive subunit CsNF-YC2 to form a nuclear NF-YB/YC complex, which binds CCAAT-box motifs to activate *CsTIC21* genes crucial for chloroplast biogenesis. However, in *tnyl3* mutants, the loss of CsNF-YB3 causes chlorosis and chloroplast development defects in cucumber.

Mechanistically, the results of this study demonstrate that CsNF-YB3 interacts with the light-responsive subunit CsNF-YC2 to form a nuclear NF-YB/YC complex, as confirmed by Y2H and split-LUC assays. These interactions suggest that CsNF-YB3 and CsNF-YC2 constitute a core transcriptional module that binds CCAAT-box motifs to activate genes crucial for chloroplast biogenesis. Recent studies have demonstrated that CsNF-YC2 directly regulates the expression of *CsTIC21*, a gene encoding a chloroplast protein translocon [27]. In line with this model, our findings further suggest that CsNF-YC2 partners with CsNF-YB3 to regulate *CsTIC21* and other chloroplast-targeted genes (Fig. 7). Collectively, our phylogenetic, expression, and interaction analyses support the idea that *CsNF-YB3* performs a distinct function within the cucumber NF-YB family. Phylogenetically, CsNF-YB3 is most closely related to CsNF-YB8:1, yet it is clearly separated from other NF-YB3-annotated paralogs (CsNF-YB3:2 and CsNF-YB3:3), indicating divergence within the NF-YB3-related group. This divergence is mirrored at the transcriptional level: *CsNF-YB3* is strongly enriched in cotyledons, which is consistent with its potential role during early seedling establishment when chloroplast development is initiated, whereas the expression of *CsNF-YB8:1* is greater in later-developing vegetative organs such as hypocotyls and roots, suggesting that the use of related *NF-YB* genes is temporally shifted. Importantly, functional specificity is further constrained by partner compatibility. In Y2H assays, CsNF-YB3 uniquely interacts with CsNF-YC2, whereas closely related CsNF-YB proteins (CsNF-YB3:2, CsNF-YB3:3, and CsNF-YB8:1) do not interact with CsNF-YC2 under the tested conditions. Taken together, these results imply that CsNF-YB3 is not simply a redundant member of a large family but rather a partner-selective, developmentally deployed NF-YB component that may act as a bottleneck for the assembly of functional NF-Y complexes required for chloroplast-related gene regulation during early development.

Thus, the loss of *CsNF-YB3* disables this transcriptional activation, leading to global photosynthesis-related gene suppression and defective chloroplast development. Notably, the severity of the *tnyl3* phenotype highlights the fundamental role of CsNF-YB3. Unlike other cucumber leaf color mutants (e.g. *v-1*, *yyl-1*, and *yl-5*), which can often survive to maturity, *tnyl3* seedlings die at early developmental stages unless supplied with exogenous sucrose, indicating the complete collapse of photoautotrophic growth [12, 14, 16]. The marked downregulation of plastid ribosomal protein genes suggests that *CsNF-YB3* may also regulate the chloroplast translation machinery, further contributing to seedling lethality. Despite these advances, several limitations remain. The direct genomic targets of the CsNF-YB3/YC2 complex have not been defined, and whether CsNF-YB3 also integrates hormone signaling or stress pathways requires further study. Chromatin immunoprecipitation sequencing (ChIP-seq) and complementation experiments will be essential to validate its direct regulatory role.

In conclusion, this work establishes CsNF-YB3 as a central transcriptional regulator of chloroplast development in cucumber, expanding the functional understanding of NF-Y complexes in plants. From an applied perspective, CsNF-YB3 represents a potential target for manipulating photosynthetic efficiency and seedling vigor, although its essentiality requires careful strategies such as partial suppression or tissue-specific regulation. Future research should focus on identifying the direct targets of CsNF-YB3, dissecting its interaction with other signaling networks, and exploring natural allelic variation for breeding programs aimed at improving light use efficiency and stress tolerance in cucurbits.

## Materials and methods

### Plant materials and growth conditions

The chlorotic mutant line *tnyl3* was identified in the M_1_ generation of a cucumber Tnt1 retrotransposon insertion mutant library derived from the North China inbred line 9930 [28]. The *tnyl3* mutant exhibits yellow cotyledons and leaves (chlorotic phenotype) and markedly stunted growth. WT cucumber inbred line 9930 (normal green leaves) was used as a control. Additionally, another inbred line, S06 (which has normal green cotyledons and is well adapted to greenhouse conditions), was used for genetic crossing. Due to the severe phenotype of *tnyl3* (mutant homozygotes do not survive beyond the development of the first true leaf), the line was maintained as heterozygotes. To investigate inheritance, heterozygous *tnyl3* plants (phenotypically normal green) were crossed with WT S06 plants. The F_1_ progeny from this cross were self-pollinated to produce an F_2_ population. Segregation of the chlorotic trait was observed in F_2_ seedlings, and individuals showing the mutant phenotype were collected for genetic analysis. For transformation assay, 9930 was used. All plants were grown in greenhouse under standard conditions (25°C, 16 hours light/8 hours dark).

### Chlorophyll content and net photosynthetic rate

Chlorophyll content was measured spectrophotometrically. Briefly, ~0.1 g of fresh leaf tissue from wild- type and *tnyl3* seedlings was sampled (in three biological replicates per genotype, each consisting of pooled leaves from multiple plants). Pigments were extracted in 10 ml of 95% ethanol in the dark at 4°C for 12 hours. The absorbance of the extract was measured at 646 and 663 nm using a UV-Vis spectrophotometer, and chlorophyll a, chlorophyll b, and total chlorophyll contents were calculated using standard formulas as described [29].

The net photosynthetic rate of leaves was assessed using a portable photosynthesis system (LI-6400XT, LI-COR Inc., USA). For this, the first true leaf of 10-day-old seedlings was measured under ambient CO_2_ and saturating light (~1000 μmol photons m^−2^∙s^−1^) conditions. At least three individual plants of each genotype were measured, and the mean assimilation rate (μmol CO_2_ m^−2^∙s^−1^) was determined.

### Transmission electron microscopy

For light microscopy of seedling morphology, seedlings were photographed with a digital camera to document cotyledon and young leaf color. For TEM, leaf samples from 10-day-old WT 9930 and *tnyl3* mutant seedlings were prepared as follows. Leaf pieces (~2 mm^2^) were cut from the middle of the cotyledons or young leaves and immediately fixed in 2.5% (v/v) glutaraldehyde in 0.1 M phosphate buffer (pH 7.2) at 4°C for 12 hours. After primary fixation, samples were rinsed in buffer and post-fixed in 1.0% osmium tetroxide at 4°C for 1.5 hours. Tissues were then dehydrated through an ethanol series of increasing concentrations (30%, 50%, 70%, 90%, 100%) and embedded in Epon resin. Ultrathin sections (~70 nm) were cut using a Leica UC-7 ultramicrotome and stained with uranyl acetate followed by lead citrate to enhance contrast. The sections were observed under a Talos L120C G2 Spirit Biotwin TEM (Thermo Fisher Scientific) at an accelerating voltage of 80 kV.

### Marker development and mapping

For initial mapping, a bulked segregant analysis (BSA) strategy was employed. Equal amounts of DNA from 20 F_2_ seedlings with chlorotic phenotype and 20 F_2_ seedlings with normal green phenotype were pooled to form mutant and WT DNA bulks, respectively. Whole-genome resequencing of parental lines and bulks were sequenced on the Illumina HiSeq 2000 platform to ~30 coverage, and the resulting reads were aligned to the cucumber reference genome (Chinese Long v4 assembly). Single-nucleotide polymorphism (SNP) calling and SNP index analysis were conducted to pinpoint regions with strong allele frequency divergence between mutant and WT bulks, following standard BSA methods[30]. In addition, insertion–deletion (InDel) polymorphisms were identified between 9930 and S06 from the resequencing data. DNA markers (InDels and SNP-derived markers) within the candidate region were developed using Primer Premier 5.0 software, and primers were synthesized by Sangon Biotech.

The candidate genes in interval were analyzed using the Cucurbit enomics Database (http://cucurbitgenomics.org/). To validate the candidate gene, gene-specific primers were designed to amplify the full-length coding sequence and genomic DNA sequence of the candidate gene from both WT 9930 and *tnyl3* mutant plants. The PCR products were Sanger-sequenced and aligned to identify mutations. Tnt1 insertion site was confirmed by whole-genome sequencing of the mutant: a large insertion in the candidate genes exon was detected in *tnyl3*. The Tnt1-specific PCR marker was then developed to genotype the presence or absence of the retrotransposon insertion in segregating individuals.

### Phylogenetic and expression analysis of CsNF-YB3

To explore the evolutionary relationships of the identified NF-YB3 protein, we performed a phylogenetic analysis. The protein sequence of CsNF-YB3 (from cucumber) and NF-YB homologs from cucumber and *Arabidopsis* were obtained from NCBI and UniProt databases. Multiple sequence alignment was conducted using ClustalW, and a phylogenetic tree was constructed with the Neighbor-Joining method in MEGA X software, with 1000 bootstrap replicates for statistical support. The gene information is listed in Supplementary Table S2.

### RNA isolation and reverse transcription quantitative PCR

For gene expression profiling, various tissues of cucumber (9930) were collected from 10-day-old seedlings, including root, hypocotyl, leaf, cotyledon. Tissues were flash- frozen in liquid nitrogen and stored at −80°C prior to RNA extraction. Total RNA was extracted using an Omini Plant RNA Kit (CW Biotech, China) with on-column DNase I treatment, following the manufactures instructions. First-strand cDNA was synthesized from 2 μg of total RNA using the HiFiScript cDNA Synthesis Kit (CW Biotech, China) and oligo(dT) primers. Reactions were set up with FastStart Universal SYBR Green Master Mix (Rox) (Roche, Shanghai) in a 20-μl volume and run on an ABI 7500 Fast Real-Time PCR System. Three biological replicates and three technical replicates were performed for each sample. The gene expression levels were calculated using the 2^−ΔΔCt^ method, normalizing to the level of expression of the cucumber *CsActin*.

To assess light responsiveness of *CsNF-YB3* and other cucumber *NF-YB* members, cucumber seedlings were grown either under continuous dark (etiolated) or normal light conditions for 7 days, and RNA was extracted from the cotyledons. qRT-PCR was performed as above to compare the genes transcript levels in light-grown vs dark-grown seedlings.

### Yeast two-hybrid assay

A Y2H experiment was conducted to test the interaction between CsNF-YB3 and candidate NF-YC partner proteins. The coding sequence of *CsNF-YB3*, *CsNF-YB3*–annotated paralogs, and *CsNF-YB8:1* were cloned into the pGBKT7 vector (Clontech), which carries the GAL4 DNA-binding domain (BD), resulting in BD-CsNF-YB3 fusion construct. Similarly, the coding sequence of a cucumber *CsNF-YC2* and *CsNF-YC9* gene (identified as a potential interactor) was cloned into the pGADT7 vector (GAL4 activation domain, AD) to create an AD–CsNF-YC2 and AD–CsNF-YC9 fusion. The specific *CsNF-YC2* selected for this assay was based on co-expression and literature evidence (CsNF-YC from cucumber family members that are expressed in seedlings). The BD and AD constructs were co-transformed into strain AH109 using the lithium acetate/PEG method. Transformants were first selected on synthetic dropout (SD) medium lacking tryptophan and leucine (SD/-Trp-Leu) to ensure both plasmids were present. After 3 days of growth at 30°C, colonies were replica-plated onto selective interaction medium lacking adenine, histidine, tryptophan, and leucine (SD/-Ade-His-Trp-Leu). Plates were incubated at 30°C for an additional 3 to 5 days. Growth of yeast on the quadruple-dropout medium indicates a positive interaction between the BD fusion and AD fusion proteins. Positive and negative controls for Y2H (provided by the kit, e.g. known interacting proteins and empty vectors) were included to verify the system functionality [31].

### Subcellular localization

The coding sequences (CDSs) of *CsNF-YC2* and *CsNF-YB3* were, respectively, integrated into the pHB-*35S::GFP* and pHB-*35S::mCherry* vector to construct pHB-*35S::CsNF-YC2-GFP,* pHB-*35S::CsNF-YC2-mCherry* and pHB-*35S::CsNF-YB3-GFP* fusion proteins, respectively. The pHB-*35S::CsNF-YC2-GFP* and pHB-*35S::CsNF-YB3-GFP* constructs were transformed into *Agrobacterium tumefaciens* GV3101, respectively. And the pHB-*35S::CsNF-YC2-mCherry* and pHB-*35S::CsNF-YB3-GFP* vectors were co-transformed into *A. tumefaciens* GV3101. Agrobacteria carrying these constructs were infiltrated into young leaves of *N. benthamiana* for transient expression. After 48 to 72 hours, the infiltrated leaf areas were observed under a Nikon ECLIPSE Ti2 confocal fluorescence microscope.

### Luciferase complementation assay (Split-LUC)

To further validate the interaction of the NF-YB/YC complex in planta, the Split-LUC assay was performed. The pC1300-CsNF-YB3-nLUC and pC1300-cLUC-CsNF-YC2 expression vectors were constructed by inserting *CsNF-YB3* and *CsNF-YC2* into pC1300-nLUC and pC1300-cLUC, respectively. pC1300-CsNF-YB3-nLUC and pC1300-cLUC-CsNF-YC2 constructs were co-transformed into *A. tumefaciens* (strain GV3101). Agrobacteria carrying these constructs were infiltrated into young leaves of *N. benthamiana* for transient expression. After 48 to 72 hours, the infiltrated leaf areas were removed and coated with LUC substrate. Then, the LUC signals were photographed using a CCD camera (Tanon5200, China).

### Dual-luciferase reporter assay

A 2.0-kb *CsTIC21* promoter fragment was cloned into a transient expression vector, pGreenII 0800-LUC, creating a *proCsTIC21:LUC* reporter. Next, the CDS sequence of *CsNF-YB3* and *CsNF-YC2* was cloned into pHB, creating effector. The reporter and effector were transformed into GV3101-pSoup, respectively. Effector and reporter were transiently co-infiltrated into cucumber cotyledons, using the pHB vector and *proCsTIC21: LUC* reporter as a negative control. There were three biological replicates and three technical replicates per effector. The dual-luciferase reporter assay was performed, as previously described [32]. The activities of ffreffy luciferase (LUC) and Renilla luciferase (REN) were detected using a Dual-Luciferase reporter gene detection kit (Yeasen Biotech, Shanghai, China).

### Cucumber transformation with CRISPR/Cas9 assay

*CsNF-YB3* was edited using the CRISPR/Cas9 system, and the DNA target was analyzed using CRISPR-P V2.0 software [33]. Guide RNA sequence was synthesized and cloned into the pKSE402 vector. *A. tumefaciens* strains EHA105 harboring the recombinant vector was used to transform the cucumber line 9930 according to the previous described methods [34]. Finally, the genomic DNA was extracted from the 9930 and the putative transgenic lines, and the *CsNT-YB3*–edited fragments were amplified and sequenced to identify the positive knock-out plants. The primers used are listed in Supplementary Table S1.

### Transcriptome sequencing and analysis

To investigate global gene expression changes caused by the *CsNF-YB3* mutation, we performed comparative RNA sequencing (RNA-Seq) on *tnyl3* and WT seedlings. Cotyledons from 10-day-old *tnyl3* mutant and 9930 WT plants were collected, with three biological replicates for each genotype. Total RNA was isolated as described above, and RNA quality was verified (RNA integrity number > 7.0). Library construction and high-throughput sequencing were conducted by Biomarker Technologies (Beijing, China). Poly(A) mRNA was enriched and reverse-transcribed to cDNA, which was then used to prepare Illumina sequencing libraries. Paired-end sequencing was performed on an Illumina NovaSeq 6000 platform, yielding ~20 million reads per sample. Raw reads were filtered to remove adapter sequences and low-quality reads, resulting in high-quality clean reads. The clean reads were aligned to the cucumber reference genome (Chinese Long v3 assembly) using HISAT2 (v2.1.0) with default parameter [35]. Transcript assembly and quantification were done using StringTie, and gene expression levels were normalized to RPKM (reads per kilobase of transcript per million mapped reads).

DEGs between *tnyl3* and wild type were identified using DESeq2. Gene counts were tested for differential expression, and those with a fold change log2 (up- or down-regulated in mutant) and a false discovery rate (FDR) < 0.01 were considered significant DEGs. Functional annotation of DEGs was performed by mapping to gene ontology (GO) and Kyoto Encyclopedia of Genes and Genomes (KEGG) databases.

## Supplementary Material

Web_Material_uhag138

## Data Availability

The data that support the findings of this study are openly available in the NCBI sequence read archive (SRA) under BioProject ID: PRJNA1401306.
