# Supplementary material for: Loss of the nuclear factor Y subunit B3 gene causes chlorosis and chloroplast development defects in cucumber
Source: Hortic Res. 2026 May 8;13(8):uhag138. doi: 10.1093/hr/uhag138 (PMC13446120; doi:10.1093/hr/uhag138)
Supplement: Supplementary file 1 [file web_material_uhag138.zip › Supplementary Figure.docx]

**Supplements Figure**

**Loss of the Nuclear Factor Y Subunit B3 Gene Causes Chlorosis and Chloroplast Development Defects in Cucumber**

Min Sheng^1,†^, Siqi Wang^2,†^, Tiefeng Song^3^, Juyong Zhao^3^, Cancan Xia ^4, 5^, Huanle He^4^, Junsong Pan^4^, Jian Pan^1,*^, Haifan Wen^4,*^

^1^ College of Horticulture, Shenyang Agricultural University, Shenyang, 110866, China;

^2^ College of Bioscience and Biotechnology, Shenyang Agricultural University, Shenyang, 110866, China;

^3^ Liaoning Academy of Agricultural Sciences, Shenyang 110161, China;

^4^ College of Agriculture and Biology, Shanghai Jiao Tong University, Shanghai, 200240, China

^5^Shanghai Pudong Agrotechnology Extension Center, Shanghai, 201201, China

^†^ These authors contributed equally to this work.

^*^ Authors for correspondence: Jian Pan (panjian@syau.edu.cn), Haifan Wen (haifanwen@sjtu.edu.cn)

**Running tittle:** *CsNF-YB3* Controls Chloroplast Development in Cucumber

Supplementary Figure S1. Phenotypes of the *tnyl3* mutant grown in MS medium with and without sucrose for two weeks, respectively. Scale bar =3 cm

Supplementary Figure S2. Light-responsive expression of cucumber *NF-YB* members. qRT-PCR comparison of *NF-YB* members transcript levels in cotyledons of dark-grown (etiolated) vs. light-grown cucumber seedlings. Bars are mean ± SD (n = 5). Asterisks indicate significant differences (****P* < 0.001, Student's *t*-test).

Supplementary Figure S3. Yeast two-hybrid assay showing that NF-YB3-annotated paralogs and CsNF-YB8:1 do not interacts with CsNF-YC2.

Supplementary Figure S4. Confocal images of tobacco epidermal cells co-expressing CsNF-YC2-mcherry and CsNF-YB3-GFP, scale bar, 50 μm.
